# Feeding challenges in larger versus smaller preterm discordant twins

**DOI:** 10.3389/fped.2026.1786672

**Published:** 2026-06-03

**Authors:** Shuyang Xu, Jing Yue, Yimin Dai, Jie Qiu

**Affiliations:** 1 Department of Neonatology, Nanjing Drum Tower Hospital, Affiliated Hospital of Medical School, Nanjing University, Nanjing, China; 2 Department of Obstetrics and Gynaecology, Nanjing Drum Tower Hospital, Affiliated Hospital of Medical School, Nanjing University, Nanjing, China

**Keywords:** birth weight discordance, full enteral feeds, necrotizing enterocolitis, preterm infant, small for gestational age, twins

## Abstract

**Background:**

This single-center retrospective cohort study aims to compare the risk of necrotizing enterocolitis (NEC) and feed intolerance between the larger and the smaller preterm twin in dichorionic (DC) and monochorionic (MC) twins with birth weight discordance (BWD).

**Methods:**

All consecutive live-born twin pairs born in the Nanjing Drum Tower Hospital between 1 January 2023, and 31 July 2025, were reviewed. BWD was defined as more than 20% difference in intertwin birth weight. Neonatal outcomes were compared between the larger and the smaller twin within each twin pair. Data on the start time of enteral feeding, time to attain full enteral feeds (FEF), delayed attainment of FEF, number of fasting episodes, maximum physiological weight loss, time to regain birth weight, the average weight growth velocity, and the frequency of breastfeeding were collected.

**Results:**

We included 30 DC twins and 35 MC twins. The smaller twin had a higher frequency of NEC than the larger twin in both the DC group (23.33% vs*.* 3.33%, *P* = 0.031) and the MC group (22.86% vs*.* 2.86%, *P* = 0.039). The incidence of respiratory distress syndrome was significantly higher (48.57% vs*.* 17.14%, *P* = 0.001) and the incidence of bronchopulmonary dysplasia was significantly lower (0.00% vs*.* 20.00%, *P* = 0.016) in the larger twin compared with the smaller twin in the MC group; however, no differences were observed in the DC group. Multivariable logistic regression results showed that gestational age and being small for gestational age (SGA) were significant influencing factors for NEC (*P* < 0.05). The smaller twin required more time to attain FEF compared with the larger twin in the DC group (1 day vs*.* 6 days, *P* = 0.002) and the MC group (5 days vs*.* 8 days, *P* = 0.000). The frequency of delays in attaining FEF and fasting in the smaller twin were significantly more than those in the larger twin in both groups (*P* < 0.05). The log-rank test revealed that the larger twin attained FEF earlier than the smaller twin both in the DC group (*P* = 0.033) and the MC group (*P* = 0.048). The results of multivariable logistic regression indicated that, in the two groups, gestational age, being SGA, and the smaller twin were significant influencing factors for delayed attainment of FEF (*P* < 0.05).

**Conclusions:**

In preterm twins with BWD, the smaller twin has an increased risk of NEC compared with the larger one, which may be attributed to the small twin being SGA. Therefore, the smaller twin needs close observation and monitoring in the early phase after birth because these preterm infants are at increased risk for feeding intolerance.

## 1 Introduction

Twin pregnancy is a health challenge, because it increases the risks of maternal and fetal complications and perinatal mortality compared with singleton pregnancies (1–3). Birth weight discordance (BWD) in twin gestations, defined as a disparity of 15%–40% in birth weights between the larger and the smaller twin, respectively (4, 5), has been associated with higher rates of adverse neonatal outcomes (6–8). Several studies have shown that the smaller twin of a pair is at a greater risk of intrauterine death, especially as growth discrepancy increases (9, 10). Therefore, early delivery is often planned to save the small twin; consequently, an increased number of twins are born prematurely and need to be admitted to the neonatal unit.

Necrotizing enterocolitis (NEC) is the most common gastrointestinal emergency in neonates. Previously established risk factors for the development of NEC include prematurity and low birth weight (BW). In a previous study, it was found that 462 (3.2%) out of 14,678 babies born before a gestational age (GA) of 32 weeks developed severe necrotizing enterocolitis, and 222 (48.1%) of these babies died (11). Although most studies have focused on presenting the association between BWD and perinatal morbidities such as respiratory distress syndrome (RDS), congenital anomalies, hyperbilirubinemia, hypoglycemia, and anemia, few studies have investigated feeding issues related to BWD, especially in larger vs. smaller preterm discordant twins.

Chorionicity represents a relevant factor influencing BWD. In dichorionic (DC) pregnancies, a different genetic growth potential may be responsible for discordant growth (12). In monochorionic (MC) pregnancies, vascular anastomoses due to the sharing of a single placenta may lead to an imbalance in intertwin transfusion and consequent asymmetric growth of twins (13–15). The different pathophysiological mechanisms of DC and MC may lead to different outcomes in newborns.

Therefore, this study aimed to investigate the incidence of NEC and other feeding conditions in twins with BWD stratified according to chorionicity. To the best of our knowledge, this study can be considered unique as it compared feeding challenges between the larger and smaller twin with BWD stratified according to chorionicity. Our results are expected to convey more accurate prognostic information to parents and doctors.

## 2 Materials and methods

### 2.1 Participants

All consecutive live-born twin pairs born in the Nanjing Drum Tower Hospital between 1 January 2023, and 31 July 2025, were reviewed. Data were collected from the records of the neonatal ward and maternal medical files. All pregnancies were dated using an early ultrasound scan. The study was approved by the ethics committee of the Nanjing Drum Tower Hospital, and the requirement for written informed consent was waived because of its retrospective design.

We excluded cases of triplet pregnancies and monochorionic monoamniotic (MCMA). Patients with twin–twin transfusion (TTTS), twin anemia-polycythemia sequence (TAPS) (16), and twin reversed arterial perfusion (TRAP) (17) who underwent no treatment were also excluded from this study. Preterm birth was defined as birth occurring before 37 completed weeks of gestation. Intertwin BWD was defined as a difference in birth weight of more than 20% and was calculated using the following formula: (IntertwinBWD=birthweightlargertwin−birthweight smallertwin)/birthweightlargertwin×100%)(18).

The following maternal and neonatal baseline characteristics were collected from medical records: maternal age at delivery, delivery mode, pregnancy-induced hypertension, gestational diabetes, premature rupture of membranes (PROM), placental abruption, gestational age at delivery, usage of antenatal steroids (defined as maternal receipt of at least one dose of dexamethasone before delivery), usage of antenatal magnesium sulfate, birth weight, sex, Apgar scores at 1 and 5 min, the proportion of neonates that were small for gestational age (SGA, defined as birth weight <10th centile), and the presence of absence or reversed end-diastolic flow (AREDF). AREDF was identified in neonates with antenatal umbilical artery Doppler showing absent/reversed end-diastolic flow on at least one occasion during pregnancy.

Enteral feeds were started using 10 mL/kg/day of expressed breast milk on day 1 if there were no contraindications to initiate feeds, such as signs of intestinal obstruction/hemodynamic instability, requiring inotropes. This was followed by slow advancement of feeds with a daily increase of 20 mL/kg/day. Breast milk was the milk of choice for feeding, and if unavailable, preterm formula (80 kcal/100 mL) was used. Breast milk was fortified when an enteral intake of 100 mL/kg/day was tolerated. Feeding strategies were modified by the neonatologist whenever there was evidence of feed intolerance or NEC.

### 2.2 Outcomes

The incidence of NEC (defined by characteristic radiographic findings accompanied by bloody stools), RDS (diagnosed based on the clinical picture of a neonate with respiratory failure requiring mechanical ventilation or continuous positive airway pressure and typical radiological findings on chest X-rays), bronchopulmonary dysplasia (BPD, diagnosed when the neonate required treatment with >21% oxygen for a postnatal age of 28 days or postconceptional age of 36 weeks), sepsis (defined as a clinically ill neonate with positive blood cultures), intraventricular hemorrhage (IVH, defined by appropriate findings on head ultrasonography), retinopathy of prematurity (ROP), patent ductus arteriosus (PDA), neonatal mortality (defined as mortality within the first 28 days after birth), major morbidity (composite outcome of death, severe RDS necessitating mechanical ventilation for more than 24 h, IVH grades 3–4, and NEC), and the duration of hospitalization were compared between the larger and the smaller twin of each twin pair.

Data collected included the start time of enteral feeding, time to attain full enteral feeds (FEF), delayed attainment of FEF, number of fasting episodes, maximum physiological weight loss, time to regain birth weight, weight gain till discharge, and the frequency of breastfeeding. The definition of enteral nutrition is as follows: (1) start time of enteral feeding: the time to start oral feeding/nasal feeding of breast milk or formula milk after birth (excluding colostrum oral care); (2) time to attain FEF: the time to attain enteral feeds of 150 mL/kg/day; (3) delayed attainment of FEF: the time to attain FEF ≥10 days (19); (4) the average weight growth velocity (GV): [1,000×ln(Wn/W1)]/(Dn−D1) after regaining birth weight. In this formula, Wn indicates weight (g) at discharge, W1 indicates birth weight (g), Dn indicates the length of hospital stay (day), and D1 indicates the time to regain birth weight (day) (20); and (5) breastfed neonates: neonates with exclusive breast milk or fortified breast milk.

### 2.3 Statistical analysis

Statistical analyses were performed using IBM SPSS Statistics Version 26.0 (IBM Corp, Armonk, NY, USA). Perinatal characteristics and neonatal outcomes of DC and MC twins were summarized using descriptive statistics. Continuous variables were reported as means ± standard deviations if normally distributed or as medians with interquartile ranges for non-normally distributed data. For categorical variables, McNemar's test was used to compare neonatal outcomes between the larger and smaller twins. For continuous variables, the paired *t*-test was used for normally distributed data, and the Wilcoxon signed-rank test was used for non-normally distributed data. Time to attain FEF was assessed using the Kaplan–Meier method, with differences between the larger and smaller twins being evaluated using the log-rank test. To account for the intrinsic correlation within twin pairs while adjusting for potential confounders, generalized estimating equations (GEEs) were used to examine potential determinants of NEC and delayed attainment of FEF. Univariable GEE models were first fitted to obtain crude odds ratios (ORs) and corresponding 95% confidence intervals (CIs). Variables with *P* < 0.05 in the univariable analysis were entered into multivariable GEE models. A *p*-value < 0.05 was considered statistically significant.

## 3 Results

**Figure 1:**
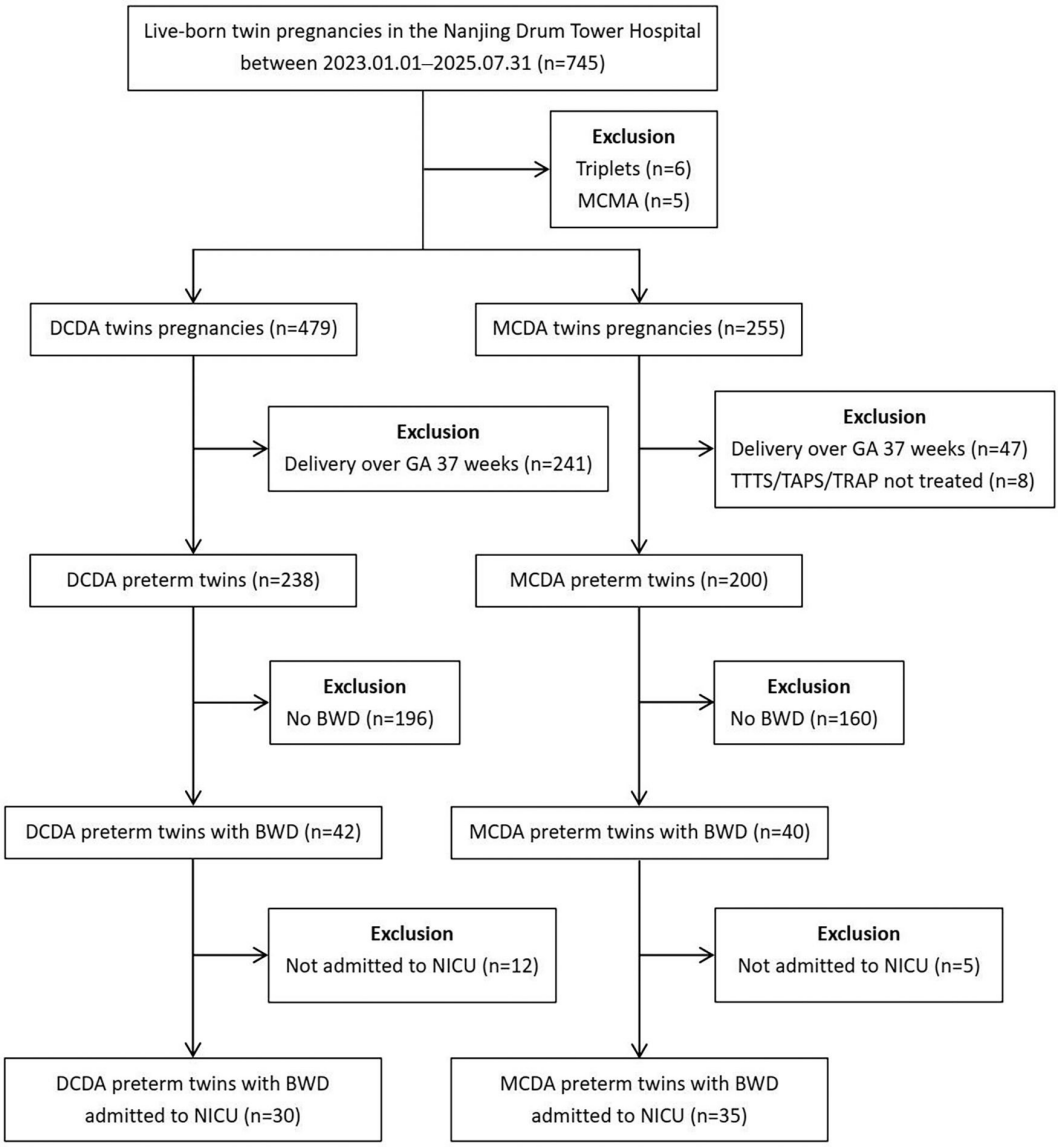
Study profile and patient flow. MCMA, monochorionic monoamniotic; DCDA, dichorionic diamniotic; MCDA, monochorionic diamniotic; GA, gestational age; BWD, birth weight discordance; TTTS, twin-twin transfusion; TAPS, twin anemia-polycythemia sequence; TRAP, twin reversed arterial perfusion; NICU, neonatal intensive care unit.

Of the 745 sets of live-born twins born at the Nanjing Drum Tower Hospital between 1 January 2023, and 31 July 2025, six sets of triplets and five sets of MCMA were excluded. Of these, 479 pairs were dichorionic diamniotic (DCDA) and 255 pairs were monochorionic diamniotic (MCDA). All twins below 37 completed weeks' gestation were identified from the admission records and twins with no BWD were excluded. After excluding preterm discordant twins who were not admitted to the neonatal intensive care unit (NICU), 30 pairs of DCDA twins and 35 pairs of MCDA twins were eligible for inclusion (Figure 1).

The maternal and neonatal baseline characteristics, which did not significantly differ between the DC group and the MC group, are summarized in Table 1. There were significant differences in birth weight, SGA, and AREDF between the larger and smaller twins, except for sex and Apgar scores.

**Table 1:** Maternal and neonatal characteristics of all BWD twin pairs.

Variable factors	DC (*n* = 30 pairs)			MC (*n* = 35 pairs)		
Maternal age (year)	31.03 ± 3.41			30.77 ± 5.13		
Caesarean delivery	28 (93.33%)			33 (94.29%)		
Pregnancy-induced hypertension	15 (50.00%)			10 (28.57%)		
Gestational diabetes	6 (20.00%)			6 (17.14%)		
PROM	2 (6.67%)			3 (8.57%)		
Placental abruption	1 (3.33%)			2 (5.71%)		
Gestational age (week)	33.75 ± 2.17			33.28 ± 1.95		
Antenatal steroids	14 (46.67%)			17 (48.57%)		
Antenatal magnesium sulfate	18 (60.00%)			14 (40.00%)		

Variable factors	Larger twin	Smaller twin	*P*	Larger twin	Smaller twin	*P*
Birth weight (g)	2,169.33 ± 408.87	1,507.50 ± 333.71	0.000	2,024.29 ± 469.83	1,438.71 ± 411.24	0.000
Male	19 (63.33%)	13 (43.33%)	0.263	17 (48.57%)	17 (48.57%)	1.000
1 min Apgar	8 (8–9)	8 (8–9)	0.392	8 (8–9)	8 (8–9)	0.942
5 min Apgar	9 (9–10)	9 (9–10)	0.348	9 (9–10)	9 (9–10)	0.257
SGA	0 (0.00%)	17 (56.67%)	0.000	0 (0.00%)	24 (68.57%)	0.000
AREDF	0 (0.00%)	6 (20.00%)	0.031	0 (0.00%)	10 (28.57%)	0.002

Data are presented as mean ± standard deviation, *n*/*N* (%), or median (IQR); PROM, premature rupture of membranes; SGA, small for gestational age; AREDF, absence or reversal of end-diastolic flow.

Neonatal complications were compared between the larger twin and the smaller twin (Table 2). The smaller twin had a higher frequency of NEC compared with the larger twin in both the DC group (23.33% vs*.* 3.33%, *P* = 0.031) and the MC group (22.86% vs*.* 2.86%, *P* = 0.039). The incidence of RDS was significantly higher (48.57% vs*.* 17.14%, *P* = 0.001) and the incidence of BPD was significantly lower (0.00% vs*.* 20.00%, *P* = 0.016) in the larger twin than in the smaller twin in the MC group; however, no significant differences were observed in the DC group. In addition, the smaller twin had a longer hospitalization than the larger twin (*P* < 0.05). There were no differences between the larger twin and the smaller twin in other outcomes.

**Table 2:** Neonatal outcomes in the larger vs. the smaller twin.

Variable factors	DC (*n* = 30 pairs)			MC (*n* = 35 pairs)		
	Larger twin	Smaller twin	*P*	Larger twin	Smaller twin	*P*
NEC	1 (3.33%)	7 (23.33%)	0.031	1 (2.86%)	8 (22.86%)	0.039
RDS	10 (33.33%)	7 (23.33%)	0.375	17 (48.57%)	6 (17.14%)	0.001
BPD	2 (6.67%)	4 (13.33%)	0.500	0 (0.00%)	7 (20.00%)	0.016
Sepsis	2 (6.67%)	3 (10.00%)	1.000	0 (0.00%)	2 (5.71%)	1.000
IVH	2 (6.67%)	4 (13.33%)	0.625	8 (22.86%)	4 (11.43%)	0.344
ROP	0 (0.00%)	0 (0.00%)	–	2 (5.71%)	0 (0.00%)	0.500
PDA	6 (20.00%)	8 (26.67%)	0.774	5 (14.29%)	9 (25.71%)	0.388
Mortality	0 (0.00%)	0 (0.00%)	–	0 (0.00%)	0 (0.00%)	–
Major morbidity^a^	4 (13.33%)	8 (26.67%)	0.344	4 (11.43%)	8 (22.86%)	0.344
Duration of hospitalization	19.61 ± 15.27	22.44 ± 16.71	0.002	19.97 ± 14.17	27.72 ± 19.63	0.000

Data are presented as *n*/*N* (%); NEC, necrotizing enterocolitis; RDS, respiratory distress syndrome; BPD, bronchopulmonary dysplasia; IVH, intraventricular hemorrhage; ROP, retinopathy of prematurity; PDA, patent ductus arteriosus;

a Composite outcome of death, severe RDS necessitating mechanical ventilation for more than 24 h, IVH grades 3–4, and NEC.

As shown in Table 3, variables that were potentially associated with the incidence rate of NEC, including gestational age, antenatal magnesium sulfate, antenatal steroids, AREDF, PDA, SGA, and being the smaller twin, were analyzed in the univariate analysis. Variables with *P* < 0.05 in the univariable analysis were entered into multivariable models. The multivariable logistic regression results of the combined risk of NEC with the above predictors showed that gestational age and being SGA were significant influencing factors for NEC (*P* < 0.05); however, no significant association was found between AREDF or being the smaller twin with NEC in both groups.

**Table 3:** Univariate and multivariate analyses of NEC in preterm twins with BWD.

Variable factors	DC (*n* = 30 pairs)						MC (*n* = 35 pairs)					
	Univariate analyses			Multivariate analyses			Univariate analyses			Multivariate analyses		
	OR	95% CI	*P*	OR	95% CI	*P*	OR	95% CI	*P*	OR	95% CI	*P*
Gestational age	0.688	0.498–0.952	0.024	0.431	0.278–0.667	0.000	0.587	0.443–0.778	0.000	0.493	0.332–0.730	0.000
Antenatal magnesium sulfate	5.552	0.651–47.330	0.117				2.065	0.565–7.554	0.273			
Antenatal steroids	4.091	0.756–22.134	0.102				2.357	0.598–9.289	0.220			
AREDF	3.887	0.772–19.569	0.100				7.333	1.583–33.966	0.011	1.975	0.296–13.179	0.482
PDA	2.236	0.517–9.673	0.281				2.273	0.500–10.323	0.288			
SGA	5.699	1.532–21.210	0.009	14.654	1.228–124.938	0.034	9.059	1.616–50.781	0.012	9.999	1.282–78.003	0.028
Smaller twin	8.826	1.340–58.149	0.024	0.352	0.009–14.424	0.581	10.074	1.109–91.536	0.040	2.174	0.085–55.389	0.638

NEC, necrotizing enterocolitis; AREDF, absence or reversal of end-diastolic flow; PDA, patent ductus arteriosus; SGA, small for gestational age; OR, odds ratio; CI, confidence interval.

The smaller twin required more time to attain FEF compared with the larger twin in both the DC group (1 day vs*.* 6 days, *P* = 0.002) and the MC group (5 days vs*.* 8 days, *P* = 0.000). The number of cases that experienced delayed attainment of FEF and fasting in the smaller twin was significantly more than those in the larger twin in both groups (*P* < 0.05). No significant differences were observed in the start time of enteral feeding, maximum physiological weight loss, time to regain birth weight, GV, and number of breastfed neonates between the larger twin and the smaller twin in both groups. These results are summarized in Table 4.

**Table 4:** Comparison of feeding condition in the larger vs. the smaller twin.

Variable factors	DC (*n* = 30 pairs)			MC (*n* = 35 pairs)		
	Larger twin	Smaller twin	*P*	Larger twin	Smaller twin	*P*
Start time of enteral feeding (day)	1 (1–1)	1 (1–1)	0.317	1 (1–1)	1 (1–1)	0.292
Time to attain FEF (day)	1 (1–4.75)	6 (1–15.25)	0.002	5 (1–10)	8 (1–21)	0.000
Delayed attain FEF (%)	4 (13.33%)	11 (36.67%)	0.016	7 (20.00%)	19 (54.29%)	0.000
Number of fasting episodes (%)	2 (6.67%)	9 (30.00%)	0.016	4 (11.43%)	11 (31.43%)	0.047
Maximum physiological weight loss (%)	−6.91 ± 2.91	−5.62 ± 3.79	0.231	−7.96 ± 3.54	−6.60 ± 4.29	0.083
Time to regain birth weight (day)	7.85 ± 5.27	6.17 ± 3.69	0.335	7.05 ± 3.15	6.61 ± 3.30	0.382
GV (g/kg/day)	8.07 ± 14.64	11.03 ± 9.58	0.098	8.69 ± 11.77	12.72 ± 13.30	0.087
Number of breastfed neonates (%)	19 (63.33%)	14 (46.67%)	0.125	24 (68.57%)	26 (74.29%)	0.500

Data are presented as median (IQR) or *n*/*N* (%); FEF, full enteral feeds; GV, the average weight growth velocity.

**Figure 2:**
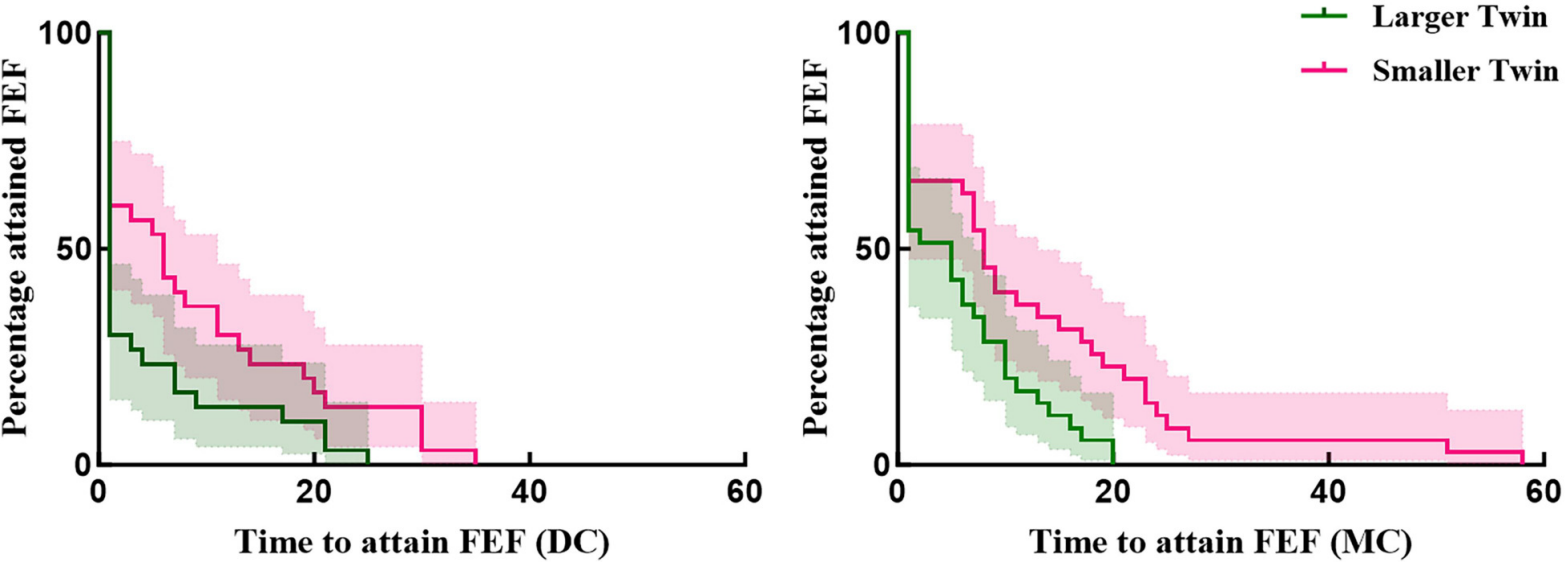
Kaplan–Meier curve showing time to attain FEF in the larger vs. the smaller twin. FEF, full enteral feed.

Kaplan–Meier survival analysis for the time to attain FEF showed a rapid decline in the curve within the first week of postnatal age, indicating that most preterm infants achieved FEF during this period. In DC twins, the earliest time to reach FEF was at 1 day postnatal, while the latest time was at 35 days postnatal. The median and mean time to attain FEF were 1 day and 4.5 ± 1.3 days (95% CI 2.0–6.9 days), respectively, in the larger twin, and were 6 days and 9.6 ± 1.9 days (95% CI 5.8–13.4 days), respectively, in the smaller twin. In MC twins, the earliest time to reach FEF was at the first postnatal day, while the latest time was at 58 days of postnatal age. The median and mean time to attain FEF were 5 days and 7.1 ± 1.6 days (95% CI 4.0–10.3 days), respectively, in the larger twin, and were 8 days and 12.2 ± 2.3 days (95% CI 7.7–16.7 days), respectively, in the smaller twin. The log-rank test revealed that the larger twin attained FEF earlier than the smaller one in both the DC group (*P* = 0.033) and the MC group (*P* = 0.048) (Figure 2).

Univariate and multivariate analyses of delayed attainment of FEF are presented in Table 5. The univariate analysis showed that gestational age, AREDF, being SGA, and the smaller twin were significantly associated with delayed attainment of FEF in both the DC group and the MC group (*P* < 0.05). The results of multivariable logistic regression indicated that, in both groups, gestational age, being SGA, and the smaller twin were significant influencing factors for delayed attainment of FEF (*P* < 0.05) but not AREDF.

**Table 5:** Univariate and multivariate analyses of delayed attainment of FEF in preterm twins with BWD.

Variable factors	DC (*n* = 30 pairs)						MC (*n* = 35 pairs)					
	Univariate analyses			Multivariate analyses			Univariate analyses			Multivariate analyses		
	OR	95% CI	*P*	OR	95% CI	*P*	OR	95% CI	*P*	OR	95% CI	*P*
Gestational age	0.496	0.345–0.714	0.000	0.342	0.214–0.545	0.000	0.571	0.434–0.751	0.000	0.390	0.233–0.652	0.000
Antenatal magnesium sulfate	1.000	0.258–3.874	1.000				2.114	0.717–6.230	0.175			
Antenatal steroids	2.053	0.521–8.082	0.304				0.500	0.029–8.555	0.632			
AREDF	4.316	1.161–16.052	0.029	1.849	0.160–21.372	0.623	11.274	1.895–67.076	0.008	2.062	0.254–16.767	0.499
PDA	1.273	0.377–4.293	0.697				1.947	0.669–5.670	0.222			
SGA	3.353	1.233–9.115	0.018	5.998	1.024–35.121	0.047	7.749	2.901–20.699	0.000	5.664	1.032–31.103	0.046
Smaller twin	3.763	1.494–9.480	0.005	5.710	1.059–30.790	0.043	10.333	3.623–29.476	0.000	11.833	1.690–82.835	0.013

NEC, necrotizing enterocolitis; AREDF, absence or reversal of end-diastolic flow; PDA, patent ductus arteriosus; SGA, small for gestational age; OR, odds ratio; CI, confidence interval.

## 4 Discussion

Our analysis of retrospective data using 30 DC preterm twins and 35 MC preterm twins with BWD revealed that, both in the DC and MC groups, smaller preterm twins had an increased risk of NEC and worse feeding conditions compared with larger preterm twins. Being SGA but not being the smaller twin was identified as the influencing factor for NEC in preterm twins with BWD; however, both SGA and being the smaller twin were significantly associated with delayed attainment of FEF.

Over the last few decades, the incidence of twin pregnancies has been rising mainly because of the significantly increased use of assisted reproductive technology and the delayed childbearing age. Decision-making in the clinical management of twin gestations depends on chorionicity and fetal growth (21–23). A systematic review and meta-analysis (10,851 twin pregnancies) showed that twins with BWD were at a higher risk of neonatal morbidity (including all cases with neurological, respiratory, or infectious morbidity, NEC, and acid-base imbalance) compared with controls (24). Correctly predicting adverse outcomes of these twins with BWD has great clinical significance for improving perinatal healthcare for twins.

Neonatal outcomes in the larger vs. the smaller twin are summarized in Table 2. In preterm MC twins with BWD but not in DC twins, being the larger twin was associated with an increased risk of RDS at birth but a reduced risk of BPD. We analyzed the potential reasons for the different neonatal respiratory outcomes between the larger twin and the smaller twin in another study.

As shown in Table 2, the smaller twin had a higher frequency of NEC in both the DC and MC groups. NEC is a common gastrointestinal emergency of preterm births and occurs in approximately 1%–8% of all neonatal intensive care unit admissions. Mortality due to NEC ranges from 15% to 30% (25). The etiology of NEC is unknown and complex. However, previous studies have reported that the incidence of NEC is strongly associated with low GA (25–27) and infants with lower BW are more likely to undergo surgery for NEC (28, 29). Considering that BW tends to decrease as GA decreases, as was also observed in this study, we did not include BW in the univariate analysis. The effect of antenatal magnesium sulfate on the incidence of NEC is still controversial (30); however, the administration of antenatal steroids has been found to decrease the incidence of NEC (31). A meta-analysis confirmed an increased incidence of NEC in preterm infants who had fetal AREDF (32). Moreover, SGA infants are considered a high-risk population for NEC, with the incidence of NEC in these infants being 5.15% (33, 34). Therefore, we assessed the association between these factors and the risk of NEC, and variables with *P* < 0.05 in the univariable analysis were entered into multivariable models. Our data confirmed that, in both groups, only GA and SGA were significant influencing factors for NEC but not being the smaller twin or having AREDF.

The results of the multivariate analysis confirmed that SGA was an independent risk factor for NEC but not being the smaller twin. Our findings are consistent with those of some previous studies that reported a higher incidence of NEC in SGA neonates compared with appropriate for gestational age (AGA, defined as birth weight 10-90th centile) neonates (35, 36). SGA, defined as a newborn with a birth weight <10th percentile for their GA (37), is often used as a surrogate for fetal growth restriction (FGR, which is the inability of the developing fetus to grow as per potential) in clinical practice. Compared with singletons, twin pregnancies have higher risks of FGR or SGA. FGR often leads to fetal hypoxia, which is the result of adverse intrauterine circumstances and may initiate cardiac output shunting away from the intestines in favor of the brain and heart with a consequent blood flow deprivation in the splanchnic district (38, 39). This process, in combination with reperfusion, may result in hypoxic-ischemic injury to the intestines, which contributes to the onset of NEC (25). Moreover, uteroplacental insufficiency may result in decreased intestinal growth (40). However, the pathogenesis of NEC in SGA neonates is still poorly understood and needs to be investigated further.

Smaller twins are already undernourished at birth, and optimum nutrition is essential for catch-up growth and development. A major challenge in postnatal management is ensuring accurate evaluation of feeding tolerance and the establishment of early FEF. Our data showed that, although the start time of enteral feeding and the breastfeeding rate were similar between the larger twin and the smaller twin, the smaller twin still had a higher rate of the delayed attainment of FEF and fasting and longer duration before attaining FEF than the larger twin (Table 4 and Figure 2). These results indicated that feeding tolerance may be worse in the smaller twin. The results of the multivariate analysis showed that both being the smaller twin and being SGA were risk factors for delayed attainment of FEF. Terefe et al. demonstrated that birth weight was one of the predictors significantly associated with time to FEF (41), which may explain that being the smaller twin was a risk factor for delayed attainment of FEF. The prolonged time to achieve FEF in SGA infants may be ascribed to a chronic prenatal intestinal hypoxic injury. Therefore, for the smaller preterm discordant twin, cautious enteral feed advancement is recommended with careful monitoring.

The influence of GA on NEC and FEF is noteworthy since preterm birth affects the optimum intrauterine growth and maturation of the gastrointestinal tract. This especially holds for SGA infants whose gut is ischemic or hypoxic. The additional effect of intrauterine growth retardation increases the risk of adverse outcomes of prematurity.

This study has several limitations. First, this is a retrospective analysis; hence, some confounding variables may not have been accounted for. Second, the groups examined were relatively small, and this may affect the validity of our negative results. Therefore, the findings of this study should be interpreted with caution.

In conclusion, in preterm twins with BWD, the smaller twin had a higher frequency of NEC compared with the larger one. Being SGA may be a suitable explanation for this observation. The smaller twin needs close observation and monitoring, especially in the early phase after birth because these preterm infants have an increased risk of feeding intolerance. Our results could guide and support antenatal parent counseling to alleviate parental anxiety. In addition, the findings of our study could guide decision-making and obstetrical management in preterm discordant twins, expectant management with close surveillance, and elective preterm birth.

## Data Availability

The original contributions presented in the study are included in the article, and further inquiries can be directed to the corresponding author/s.
